# Clinical signs predictive of severe illness in young Pakistani infants

**DOI:** 10.1186/s13104-021-05486-y

**Published:** 2021-02-24

**Authors:** Shahira Shahid, Shiyam Sunder Tikmani, Kanwal Nayani, Ayesha Munir, Nick Brown, Anita K. M. Zaidi, Fyezah Jehan, Muhammad Imran Nisar

**Affiliations:** 1 Department of Pediatrics and Child Health, Aga Khan University, Karachi, Pakistan; 2 Department of Community Health Sciences, Aga Khan University, Karachi, Pakistan; 3 Salisbury District Hospital, Salisbury, UK; 4 Bill & Melinda Gates Foundation, Seattle, USA

**Keywords:** Young infants, Clinical signs, Severe illness requiring hospitalization, Pakistan, Community

## Abstract

**Objective:**

Early detection of specific signs and symptoms to predict severe illness is essential to prevent infant mortality. As a continuation of the results from the multicenter Young Infants Clinical Signs and Symptoms (YICSS) study, we present here the performance of the seven-sign algorithm in 3 age categories (0–6 days, 7–27 days and 28–59 days) in Pakistani infants aged 0–59 days.

**Results:**

From September 2003 to November 2004, 2950 infants were enrolled (age group 0–6 days = 1633, 7–27 days = 817, 28–59 days = 500). The common reason for seeking care was umbilical redness or discharge (29.2%) in the 0–6 days group. Older age groups presented with cough (16.9%) in the 7–27 age group and (26.9%) infants in the 28–59 days group. Severe infection/sepsis was the most common primary diagnoses in infants requiring hospitalization across all age groups. The algorithm performed well in every age group, with a sensitivity of 85.9% and specificity of 71.6% in the 0–6 days age group and a sensitivity of 80.5% and specificity of 80.2% in the 28–59 days group; the sensitivity was slightly lower in the 7–27 age group (72.4%) but the specificity remained high (83.1%).

## Introduction

Neonatal period accounted for approximately 45% of all under-five mortality in 2015 particularly in developing countries [1, 2]. A multi-country study called the Young Infant Clinical Signs Study (YICSS) was undertaken in developing countries (Dhaka, Bangladesh; La Paz, Bolivia; Kumasi, Ghana; Chandigarh, India; Delhi, India; Durban, South Africa and Karachi, Pakistan). It assessed predictive values of several symptoms and signs as elicited by a primary health worker in determining severe illness requiring hospitalization by using a unified protocol [3]. The study analyzed data from 3177 infants aged 0–6 days and 5712 infants aged 7–59 days and an initial set of 12 symptoms reduced to 7 key indicators of disease severity. The most important signs and symptoms were noted to be: history of difficulty feeding; convulsions; movement only when stimulated; respiratory rate of 60 breaths per minute or more; severe chest indrawing and temperature of 37.5 °C or more or below 35.5 °C [3–7]. These results were used to update the Integrated Management of Childhood Illness (IMCI) guidelines [8].

This paper reports experience from the contributory study undertaken by the Aga Khan University in Karachi, Pakistan, where, unlike all other sites, only data from the community was used. We report the data from 1633 infants aged 0–6 days, 817 infants aged 7–27 days and 500 infants aged 28–59 days, the largest contribution to the YICSS by a single country, with the sample size for neonates less than a week old in our study being more than half of the total sample size of neonates aged 0–6 days in the main study. The objective of this study is to validate the clinical signs and symptoms observed in infants to identify severe illness and predict hospital admissions.

## Main text

### Methods

The study was undertaken in three field sites in Karachi, Pakistan: Bilal Colony, Rehri Goth and Ibrahim Hyderi from 10th September 2003 to 13th November 2004. Two of these are peri-urban sites with Primary Healthcare Centers (PHC) run by the Aga Khan University Hospital (AKUH), and a third (Bilal Colony) is a densely populated urban squatter settlement. A uniform methodology was used at all study sites, details of which are described in the main YICSS paper [3]. All infants under the age of 60 days who were either self-referred to our center or referred by Community Health Workers (CHW) during community surveillance were enrolled after written informed consent was taken from the parent/guardian. Enrollment took place 4 days a week, i.e. Monday, Tuesday, Thursday, and Friday from 9 am to 3 pm each day. Infants who lived outside the catchment area, sick infants with a history of hospitalization in the previous 2 weeks, along with those who had a lethal malformation or those requiring emergent treatment, and those whose parents or guardians did not consent were excluded.

#### Study procedure

Young infants were first screened by a trained LHV (Lady Health Visitor) (Study Person A) after determining eligibility and taking informed consent. Socio-demographic details and clinical signs were recorded from a pre-specified list on form A. The infant was then referred to an experienced pediatrician (Study person B) who was blinded to the assessment of the LHV. The pediatrician determined the need for immediate referral of the infants to a tertiary care hospital, the National Institute of Child Health (NICH), based on their clinical presentation. Infant pulse and oximetry were also performed. Training sessions for the LHVs and pediatricians were conducted in which clinical signs were demonstrated using videos. All infants who were not referred for admission were followed-up within 48 h during which they were re-evaluated for any improvement or deterioration. In case a follow-up visit was not made, attempts were made to contact the child’s parent or guardian using telephone otherwise a home visit was made. A review committee of three pediatricians reviewed records of 20% of all admitted infants and 10% of all infants who were not admitted. The feedback was provided to Study Person B.

#### Data entry, cleaning, and management

All forms were checked for completion and correctness and then entered into an Epi-Data database (V.2.1, Epidata Association, Odense Denmark). Data files were double checked by the international data coordination center in Melbourne, Australia, for consistency and quality.

#### Statistical methods

Using the methods described in the main paper, we replicated the analysis for 0–6-day age group in Pakistani infants. Our primary outcome was the classification of severe illness requiring hospitalization by Study Person B. Univariate analysis of the association between the primary outcome and individual clinical signs and symptoms was conducted and Odds Ratio (OR) with 95% Confidence Intervals (CI) were calculated. Since decision to refer infants with jaundice is based on the level of hyperbilirubinemia and not just signs and symptoms alone, these children were included in the non-severe illness category [3]. We calculated sensitivity and specificity of individual signs in the WHO seven sign algorithm [8]. The algorithm derived from the 0–6 days group was then used to calculate sensitivity and specificity for 7–27- and 28–59-days age group. All analysis was done using Stata version 12.0.

#### Ethical clearance

The study was approved by the Ethics Committee at Aga Khan University Hospital and the WHO Ethical Research Committee.

### Results

A total of 3011 infants were triaged, out of which 2950 were enrolled. Of all the three age groups, 329 infants required hospitalization, and 92 were admitted. The flow of infants in the study is given in Additional file 1: Figure S1.

Table 1 shows the baseline characteristics of the enrolled infants, 55% of the infants were aged 0–6 days, 28% were aged 7–27 days and 17% were aged 28–59 days. Majority of the infants were delivered at home (78%). The most common reason for seeking care in the 0–6 days group was umbilical redness or discharge (29.2%), followed by excessive crying (11.1%). In the older age group, 16.9% infants in the 7–27 age group presented with cough, 14.1% presented with fever and 9.7% presented with diarrhea. The most common primary diagnosis (assessed by the pediatrician) in the 0–6 days group was localised infection (including skin, eye, oral and umbilical infections) 44.7%. In the older age group, the most common primary diagnosis was found to be upper respiratory tract infection (URTI), with 33.6% diagnosed infants in the 7–27 days group and 32.6% in the 28–59 days group (Table 2).

**Table 1:** Baseline clinical characteristics of study groups

Characteristics	Number of infants N = 2950	
0–6 days	1633	(55%)
7–27 days	817	(28%)
28–59 days	500	(17%)
Male	1467	(50%)
Exclusive breastfeeding	1578	(54%)
Vital signs		
Pulse (> 160 bpm)	30	(1%)
Pulse oximetry (< 95%)	513	(17%)
Obstetric history		
Home delivery	2298	(78%)
Delivered by skilled attendant	720	(24%)
Low Birth Weight (< 2500 g)	942	(32%)
Parity > 0	520	(18%)
Gestational age (< 37 weeks)	59	(2%)
Maternal tetanus toxoid	645	(22%)
Antenatal care (3 visits or more)	624	(21%)
Diabetes during pregnancy	5	(0%)
Anemia during pregnancy	1049	(36%)
UTI during pregnancy	35	(1%)
Fever at the time of delivery	382	(13%)
Prolonged labor	191	(7%)
Prolonged rupture of membranes	170	(6%)
Vaginal delivery	2878	(98%)

Data are in n (%) or mean (SD)

**Table 2:** Primary diagnosis by age group for severe illness (assessed by pediatrician) and need for hospital management

	Required Admission			Did not require admission			Total infants in each age category			
Diagnosis	0–6 days, n = 220	7–27 days, n = 68	28–59 days, n = 41	0–6 days, n = 1413	7–27 days, n = 749	28–59 days, n = 459	0–6 days, n = 1633	7–27 days, n = 817	28–59 days, n = 500	Total 2950
Prematurity/LBW	47 (21.4)	9 (13.2)	4 (9.8)	141 (9.9)	38 (5.1)	20 (4.4)	188 (11.5)	47 (5.7)	24 (4.8)	259 (8.7)
Birth Asphyxia	23 (10.4)	0 (0.0)	0 (0.0)	34 (2.4)	5 (0.7)	2 (0.4)	57 (3.5)	5 (0.6)	2 (0.4)	64 (2.2)
Birth Injury	0 (0.0)	0 (0.0)	0 (0.0)	1 (0.1)	2 (0.3)	2 (0.4)	1 (0.06)	2 (0.2)	2 (0.4)	5 (0.2)
Congenital Malformations	2 (0.9)	1 (1.5)	0 (0.0)	1 (0.1)	1 (0.1)	1 (0.2)	3 (0.18)	2(0.2)	1 (0.2)	6 (0.2)
Hyperbilirubinemia, physiological jaundice	35 (15.9)	5 (7.4)	5 (12.2)	130 (9.2)	43 (5.7)	14 (3.1)	165 (10.1)	48 (5.9)	19 (3.8)	232 (7.9)
Severe infection (sepsis, pneumonia, meningitis)	96 (43.6)	50 (73.5)	26 (63.4)	0 (0.0)	2 (0.3)	1 (0.2)	96 (5.9)	52 (6.4)	27 (5.4)	175 (5.9)
URTI, mild ARI	0 (0.0)	0 (0.0)	0 (0.0)	151 (10.7)	275 (36.7)	163 (35.5)	151 (9.2)	275 (33.6)	163 (32.6)	589 (19.9)
Localized infections (skin, eye, umbilical, oral)	3 (1.4)	2 (2.9)	2 (4.9)	727 (51.4)	243 (32.4)	157 (34.2)	730 (44.7)	245 (29.9)	159 (31.8)	1134 (38.4)
Diarrhea, dysentery, persistent diarrhea, dehydration	0 (0.0)	1 (1.5)	1 (2.4)	25 (1.8)	68 (9.0)	70 (15.3)	25 (1.5)	69 (8.4)	71 (14.2)	165 (5.6)
Colic, gastrocolic reflex, regurgitation, constipation	0 (0.0)	0 (0.0)	0 (0.0)	39 (2.8)	29 (3.9)	13 (2.8)	39 (2.38)	29 (3.5)	13 (2.6)	81 (2.7)
Others	14 (6.4)	0 (0.0)	3 (7.3)	163 (11.5)	38 (5.1)	13 (2.8)	177 (10.8)	38 (4.6)	16 (3.2)	231 (7.8)
Well baby	0 (0.0)	0 (0.0)	0 (0.0)	1 (0.1)	5 (0.7)	3 (0.7)	1 (0.06)	5 (0.6)	3 (0.6)	9 (0.3)

Data is shown as n (%)

Additional file 1: Table S1 describes the individual signs and symptoms in predicting the need for hospitalization in each age group. History of fever, history of no cry at birth, respiratory rate > 60 breaths per minute and redness and pus around the umbilicus emerged as the most prevalent signs in the 0–6 days group. In the older groups, history of fever remained the most prevalent sign. We compared the point estimates of odds ratios for different signs and symptoms. Among the most common signs (prevalence more than 10%), signs such as moving only on stimulation, lethargy, severe chest indrawing, and nasal flaring appeared as having the greatest odds of predicting severe illness requiring hospitalization in the 0–6 days group, while in the older age groups, movement only on stimulation and prolonged capillary refill had higher odds as compared to other signs and symptoms in the group.

We used the WHO algorithm for young infants for evaluating the effectiveness of signs in the Pakistani population [8]. As reported in the main paper, we reported an identical sensitivity of 85.9% and specificity of 71.6% in the 0–6-day age group. The same model was applied to the older age groups. In the 7–27 days age group, the algorithm had a sensitivity of 72.4% and a specificity of 83.1% while in the 28–59 days group, it had a sensitivity of 80.5% and a specificity of 80.2%. Sensitivity and specificity of each sign in predicting the need for hospitalization in young Pakistani infants is given in Table 3.

**Table 3:** Sensitivity and specificity of individual signs, and the 7-sign algorithm for identification of the sick infant requiring hospital admission, for each age group

Signs	Ages 0–6 days n = 1633		Ages 7–27 days n = 817		Ages 28–59 days n = 500	
	Sensitivity (95% CI)	Specificity (95% CI)	Sensitivity (95% CI)	Specificity (95% CI)	Sensitivity (95% CI)	Specificity (95% CI)
History of difficult feeding	26.0 (19.7, 33.0)	97.2 (96.2, 98.0)	24.1 (13.9, 37.2)	96.6 (95.0, 97.8)	15.0 (5.7, 29.8)	93.7 (91.1, 95.7)
Movement only when stimulated	22.2 (17.5, 27.5)	99.7 (99.4, 99.8)	20.7 (11.2, 33.4)	99.6 (98.8, 99.9)	7.3 (1.5, 19.9)	99.8 (98.8, 100.0)
Temperature < 35.5	13.0 (8.5, 18.7)	98.0 (97.1, 98.7)	5.2 (1.1, 14.4)	98.9 (97.9, 99.5)	2.4 (0.1, 12.9)	99.3 (98.1, 99.9)
Temperature ≥ 37.5	24.7 (18.6, 31.7)	90.8 (89.2, 92.3)	27.6 (16.7, 40.9)	96.3 (94.6, 97.5)	12.2 (4.1, 26.2)	96.0 (93.8, 97.6)
Respiratory Rate > 60	51.4 (43.9, 58.8)	81.6 (79.5, 83.5)	46.6 (33.3, 60.1)	92.1 (89.9, 93.9)	56.1 (39.7, 71.5)	92.2 (89.3, 94.4)
Severe chest indrawing	12.4 (8.0, 18.1)	99.7 (99.2, 99.9)	24.1 (13.9, 37.2)	100.0 (99.5, 100.0)	39.0 (24.2, 55.5)	97.6 (95.8, 98.8)
History of convulsions	9.7 (5.9, 14.9)	99.5 (99.0, 99.8)	1.7 (0.0, 9.2)	98.7 (97.6, 99.4)	9.8 (2.7, 23.1)	98.5 (96.9, 99.4)
7-sign algorithm	85.9 (80.1, 90.6)	71.6 (69.2, 74.0)	72.4 (59.1, 83.3)	83.1 (80.3, 85.7)	80.5 (65.1, 91.2)	80.2 (76.2, 83.7)

### Discussion

Our results show that the WHO IMCI algorithm for predicting severe illness is effective in young Pakistani infants aged 0-59 days. The 7-sign algorithm (history of difficult feeding, movement only when stimulated, temperature < 35.5, temperature ≥ 37.5, respiratory rate > 60, severe chest indrawing and history of convulsions) derived from infants aged 0–6 days is predictive of severe illness not only in that age group, but is also applicable to older infants aged 7–27 and 28–59 days. This study was conducted as part of a multicenter trial to assess the signs and symptoms of severe illness in Karachi, Pakistan. The study sites were low-income urban and peri-urban areas with limited access to primary healthcare facilities.

The most common primary diagnosis in infants requiring hospitalization was severe infections/sepsis, with a prevalence of 44% in the 0–6 days group, 74% in the 7–27 days group and 61% in the 28–59 days group therefore early detection of warning signs by health workers in neonates and infants is crucial [9]. The IMCI criteria includes seven signs and the presence of any clinical sign is considered to be suspicious for a serious bacterial illness [8]. This is especially important for infants aged 0–6 days, since most of the neonatal deaths take place in the first week of life [2]. The signs usually included in the algorithm if used alone have a limited prediction value for referral because they are mostly accompanied by other common signs and symptoms. As a result, they are difficult to retain and teach because they are infrequently seen alone [6, 10]. In our univariate analysis, we focused on signs and symptoms with a minimum of 5% prevalence. Having an acceptable level of sensitivity and specificity, and consisting of only seven signs, the algorithm proves to be a simpler criterion of recognizing severe illness in neonates. It is quick and easy to teach and retain as far as the health workers in the community settings are concerned.

An important strength of our study was that it included a relatively higher number of infants aged less than 7 days which helped in generating signs and symptoms for this age category with a greater accuracy and precision as compared to other similar studies in different countries that had a lower number [3]. Moreover, our study was conducted in a community level setting with no health insurance or tertiary care coverage as compared to the same study in other centers [3]. Our results show that the seven-sign algorithm is indeed effective in predicting the need for hospitalization in the community setting, where such a technique proves invaluable in face of the dearth of resources and accessibility, as well as high mortality rate.

### Conclusion

With relevant guidelines in place, LHVs (Lady Health Visitors) can be trained to recognize signs and symptoms of severe illnesses in infants, thereby contributing significantly towards the improvement of the infant and neonatal outcomes in a low and middle-income country like Pakistan, as well as globally.

## Limitations

- Diseases in the early neonatal period are rarely reported when it comes to rural or semi-urban areas with community level health facilities, despite their immense significance in reducing the childhood mortality if detected and managed early [11–13]. To avoid this limitation, we conducted regular community surveillance to ensure maximum participation, and to avoid missing any possible cases of severe illness.
- Another limitation in our study was that the decision for referral was made by the physician purely based on history and clinical examination and was not supported by laboratory tests. Even though tests were conducted wherever indicated, the results did not affect the decision for hospital admission. In this case, there is a possibility that some cases of severe illness were missed simply because the child had not developed symptoms, or that the symptoms were not very apparent.
- We excluded infants who were previously sick and were referred by another healthcare professional in order to mimic the community population presenting to a primary healthcare facility and this led to lesser number of sick infants requiring hospital admissions.
- A separate analysis should be conducted in order to study indicators of jaundice and hyperbilirubinemia, as that too is an important cause of neonatal morbidity.
- Although it’s been a long time since this study was completed and primary results were published, (primary data for this study was collected between 2003 and 2004) [3], we still think that these site specific results are of considerable value for similar low middle income settings. They would additionally be helpful for researchers updating systematic review and metanalysis in this field.

## Supplementary information

**Additional file 1.** Supplementary Figure and Table

## Data Availability

The datasets used and/or analyzed during the current study are available from the corresponding author on reasonable request.

## Abbreviations

YICSS: Young Infants Clinical Signs and Symptoms
PHC: Primary Health Center
AKUH: Aga Khan University, Hospital
IMCI: Integrated Management of Childhood Illnesses
WHO: World Health Organization
CHW: Community Health Worker
LHV: Lady Health Visitor
NICH: National Institute of Child Health

## References

[1] WHO, Global health observatory data-neonatal mortality. 2015.

[2] WHO. Newborns: reducing mortality. http://www.who.int/mediacentre/factsheets/fs333/en/. 2016.

[3] Young Infants Clinical Signs Study (2008). Clinical signs that predict severe illness in children under age 2 months: a multicentre study. Lancet.

[4] Mazzi E (2010). Clinical signs predicting severe illness in young infants (< 60 days) in Bolivia. J Trop Pediatr.

[5] Yeboah-Antwi K (2008). Clinico-epidemiological profile and predictors of severe illness in young infants (0–59 days) in Ghana. Ann Trop Paediatr.

[6] Deorari AK (2007). Clinicoepidemiological profile and predictors of severe illness in young infants (< 60 days) reporting to a hospital in North India. Indian Pediatr.

[7] Jeena PM (2008). Clinical profile and predictors of severe illness in young South African infants (< 60 days). S Afr Med J.

[8] WHO, Integrated management of childhood illnesses-Chart Booklet. 2014.

[9] WHO, Newborn death and illness. 2011.

[10] Kumar V, Singhi S (1994). Predictors of serious bacterial infection in infants up to 8 weeks of age. Indian Pediatr.

[11] Duke T (2005). The management of sick young infants at primary health centres in a rural developing country. Arch Dis Child.

[12] English M (2003). Causes and outcome of young infant admissions to a Kenyan district hospital. Arch Dis Child.

[13] Dagan R (1985). Identification of infants unlikely to have serious bacterial infection although hospitalized for suspected sepsis. J Pediatr.

