# Supplementary material for: Clinical signs predictive of severe illness in young Pakistani infants
Source: BMC Res Notes. 2021 Feb 24;14:71. doi: 10.1186/s13104-021-05486-y (PMC7903754; doi:10.1186/s13104-021-05486-y)
Supplement: Supplementary file 1 — Additional file 1. Supplementary Figure and Table [file 13104_2021_5486_MOESM1_ESM.docx]

**Figure s1 – Participant flow** **chart**

Admitted

N=8

Refused admission

N=33

Sent home

N=749 (91.7%)

Refused admission

N=44

Admitted

N=24

Improved on follow up

N=741

Worsened and admitted/died

N=2

Loss to follow up

N=6

Improved on follow up

N=451

Worsened and admitted/died

N=3

Loss to follow up

N=5

Severe illness requiring hospitalization

N=41 (8.2%)

Severe illness requiring hospitalization

N=68 (8.3%)

Died

N=1

Discharged

N=7

Died

N=1

Discharged

N=23

Sent home

N=459 (91.8%)

Loss to follow up

N=19

Worsened and admitted/died

N=14

Improved on follow up

N=1380

Refused admission

N=160

Admitted

N=60

Died

N=8

Discharged

N=52

Sent home

N=1413 (86.5%)

Severe illness requiring hospitalization

N=220(13.5%)

Age Group I (0-6 days)

N=1633

Age Group III (28-59 days)

N=500

Age Group II

(7-27 days)

N=817

Total enrolled

N=2950

Total triaged

N=3011

Excluded (N=61)

Refused consent = 48

Living outside catchment area = 2

Came with same illness = 11

***Table s1: Signs Predicting serious illness requiring urgent hospital management in young infants aged 0-59 days.***

| Diagnosis | 0-6 days | | 7-27 days | | 28-59 days | |
| --- | --- | --- | --- | --- | --- | --- |
|  | P | OR (95% CI) | P | OR (95% CI) | P | OR (95% CI) |
| History of fever | 22.9 | 2.1 (1.5,2.9) | 37.6 | 3.2 (1.8, 5.5) | 43.5 | 1.4 (0.7,2.6) |
| History of difficult feeding | 5.4 | 12.3 (7.7, 19.4) | 4.9 | 8.9 (4.3,18.3) | 7.0 | 2.6 (1.0,6.7) |
| Not feeding well | 5.2 | 8.0 (3.5,18.3) | 2.2 | 9.6 (3.0,31.0) | 0.7 | 24.8 (2.2,281.7) |
| Movement only when stimulated | 3.3 | 101.1(39.5,258.2) | 1.8 | 65.6(17.9,240.5) | 0.8 | 36.2 (3.6,356.1) |
| Lethargic | 5.0 | 22.4 (13.6,36.7) | 3.8 | 25.7 (11.8,56.3) | 2.2 | 15.5 (4.5,53.5) |
| Restless and Irritable | 5.3 | 4.2 (2.5,6.7) | 3.9 | 4.8 (2.1,11.4) | 8.0 | 11.6 (5.4,24.5) |
| Temp. < 35.5◦C | 3.3 | 7.3 (4.1,12.8) | 1.4 | 5.1 (1.3,19.8) | 0.8 | 3.8 (0.4,37.3) |
| Temp. ≥ 37.5◦C | 10.9 | 3.2 (2.2,4.7) | 5.5 | 9.8 (4.9,19.5) | 4.7 | 3.3 (1.2,9.5) |
| Prolonged capillary refill | 2.9 | 14.1 (7.7,25.6) | 0.9 | 35.7 (6.7,188.4) | 1.4 | 78.5 (9.2,670.4) |
| RR ≥ 60/min | 22.2 | 4.6 (3.4,6.4) | 10.6 | 10.1 (5.6,18.1) | 11.8 | 15.0 (7.4,30.3) |
| Nasal flaring | 0.2 | 23.8 (2.4,230.5) | 0.7 | 28.0 (5.0,156.5) | 0.6 | - |
| Grunting | 2.0 | 12.6 (6.1,26.0) | 3.4 | 7.1 (3.1,16.6) | 12.4 | 7.4 (3.7,14.7) |
| Cyanosis | 0.6 | - | 0.1 | - | 0.2 | - |
| Severe chest drawing | 1.7 | 40.9 (15.3,109.2) | 1.7 | - | 5.4 | 26.0 (10.9,62.0) |
| History of no cry at birth | 22.6 | 2.9 (2.1,4.1) | 21.5 | 0.9 (0.5,1.8) | 20.0 | 1.7 (0.8,3.5) |
| History of convulsions | 1.5 | 22.2 (9.1,53.9) | 1.4 | 1.3 (0.1,10.4) | 2.2 | 6.9 (1.9,24.8) |
| Bulging fontanelle | 0.4 | 15.9 (2.9,87.7) | 0.1 | - | 0.4 | 11.5 (0.7,186.5) |
| History of diarrhea | 5.2 | 0.8 (0.4,1.6) | 19.1 | 1.6 (0.9,3.1) | 23.4 | 0.5 (0.2,1.3) |
| History of diarrhea > 14 days | - | - | 1.4 | - | 2.4 | - |
| History of blood in stool | 0.1 | - | 0.0 | - | 0.4 | - |
| Sunken eyes | 0.1 | - | 1.0 | 8.2 (1.9,35.3) | 2.2 | 1.1 (0.1,8.9) |
| Reduced skin turgor | 3.1 | 3.1 (1.6,5.8) | 2.3 | 10.8 (4.2,28.2) | 1.6 | 12.3 (2.2,51.2) |
| History of feeding problem since birth | 3.8 | 12.2 (7.1,20.7) | 1.7 | 10.8 (3.6,32.4) | 0.8 | 37.1 (3.7,365.9) |
| Many or severe skin pustules | 4.5 | 0.6 (0.2,1.4) | 18.7 | 1.0 (0.5,2.0) | 4.4 | - |
| Purulent drainage from eyes | 12.2 | 0.7 (0.4,1.2) | 15.3 | 1.0 (0.5,2.1) | 9.2 | 0.7 (0.2,2.5) |
| Purulent ear discharge | 0.1 | - | 0.2 | - | 1.4 | - |
| Redness around umbilicus | 25.1 | 0.9 (0.7,1.4) | 2.2 | - | 0.8 | - |
| Pus from umbilicus | 33.4 | 0.5 (0.3,0.6) | 23.0 | 0.8 (0.4,1.6) | 4.8 | 0.4 (0.1,3.6) |
| Stiff limbs | 0.7 | 91.4 (11.7,712.8) | 0.1 | - | 0.2 | - |
| Hardening of skin | 7.3 | 0.7 (0.4,1.4) | 7.8 | 0.2 (0.0,1.4) | 1.2 | - |
| Abdominal distension | 0.6 | 8.0 (2.2,27.9) | 2.1 | 4.2 (1.3,13.4) | 1.6 | 1.6 (0.2,13.4) |

***P=Prevalence, OR=Odds Ratio, CI=Confidence Interval, Temp. = Temperature, RR=Respiratory Rate***
